# The elusive transcriptional memory trace

**DOI:** 10.1093/oons/kvac008

**Published:** 2022-06-16

**Authors:** Beatriz Gil-Marti, Celia G Barredo, Sara Pina-Flores, Jose Luis Trejo, Enrique Turiegano, Francisco A Martin

**Affiliations:** Molecular Physiology of Behavior Laboratory, Department of Molecular, Cellular and Developmental Neurobiology, Cajal Institute, Spanish National Research Council (CSIC), 28002 Madrid, Spain; Department of Biology, Autonomous University of Madrid, 28049 Madrid, Spain; Molecular Physiology of Behavior Laboratory, Department of Molecular, Cellular and Developmental Neurobiology, Cajal Institute, Spanish National Research Council (CSIC), 28002 Madrid, Spain; Molecular Physiology of Behavior Laboratory, Department of Molecular, Cellular and Developmental Neurobiology, Cajal Institute, Spanish National Research Council (CSIC), 28002 Madrid, Spain; Neurogenesis of the Adult Animal Laboratory. Department of Translational Neuroscience, Cajal Institute, Spanish National Research Council (CSIC), 28049, Madrid, Spain; Department of Biology, Autonomous University of Madrid, 28049 Madrid, Spain; Molecular Physiology of Behavior Laboratory, Department of Molecular, Cellular and Developmental Neurobiology, Cajal Institute, Spanish National Research Council (CSIC), 28002 Madrid, Spain

**Keywords:** molecular signature, trace, learning, memory, transcriptomics, engram

## Abstract

Memory is the brain faculty to store and remember information. It is a sequential process in which four different phases can be distinguished: encoding or learning, consolidation, storage and reactivation. Since the discovery of the first *Drosophila* gene essential for memory formation in 1976, our knowledge of its mechanisms has progressed greatly. The current view considers the existence of engrams, ensembles of neuronal populations whose activity is temporally coordinated and represents the minimal correlate of experience in brain circuits. In order to form and maintain the engram, protein synthesis and, probably, specific transcriptional program(s) is required. The immediate early gene response during learning process has been extensively studied. However, a detailed description of the transcriptional response for later memory phases was technically challenging. Recent advances in transcriptomics have allowed us to tackle this biological problem. This review summarizes recent findings in this field, and discusses whether or not it is possible to identify a transcriptional trace for memory.

## INTRODUCTION

**Figure 1:**
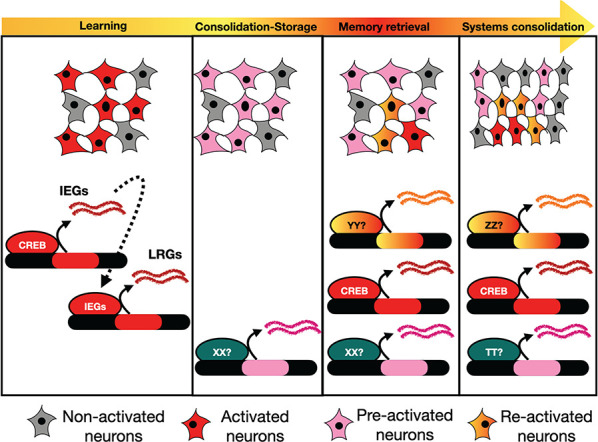
Current hypothesis of cellular engrams. Experience induces neuronal activity in a particular cellular network (engram), for instance in the hippocampus. In such neurons, a wave of CREB-dependent immediate early genes (IEGs) transcription also causes transcriptional activity in late response genes (LRGs). After learning, consolidation/storage takes place, probably mediated by a set of transcripts. In memory retrieval, only some of pre-activated neurons are re-activated (i.e. memory retrieval) but also novel activated neurons join the memory engram (reconsolidation). In the former case, IEGs should coexist with a wave of a re-activation transcriptional program. After systems consolidation, memory retrieval triggers mainly the memory engram in another structure (such as the prefrontal cortex), which also incorporates new neurons.

Memory has a very simple definition: it is the faculty of encoding, storing and retrieving information [48]. Until recently, we did not have the appropriate molecular tools to study memory mechanisms in detail [29]. The most accepted hypothesis is that experience triggers the activation of a sparse ensemble of neurons known as cellular engrams, which can comprise several brain regions (Fig. 1) [26]. It is assumed that within this ensemble of neurons activated by the experience we can find the memory engram: the physical substrate of memory would be a cluster of neurons that fire together when the same stimulus was presented again, leading to memory retrieval (Fig. 1). This clustered activity would occur due to modifications in the excitability and/or circuitry, which persisted in time [18]. The memory engram is thought to be maintained by reinforced synapses created *de novo* or already existing. Actually, it correlates with observed changes in synaptic plasticity and the phenomenon of long-term potentiation (LTP) (see below) [40]. The importance of the synapse in the formation of long-term memory (LTM) has driven to some authors to propose it as the unit of memory, defining the synaptic engram [20]. In any case, synapse modifications are achieved by local protein synthesis and degradation, *mRNA* transport, *RNA*-binding protein activity, epigenetic modifications and gene transcription [3, 41].

LTP is widely accepted as the neuronal correlate of learning [55]. LTP is the strengthening of synapses following repeated synaptic activity, reflected in changes in the electrophysiological response [1]. An initial early-transient LTP could be transformed into a late-persistent LTP response within a temporal window, which would correspond to short- and LTM formation, respectively [5]. This might be achieved by a synaptic tagging that would specifically capture plasticity-related proteins (PRPs). These PRPs required *de novo* synthesis of protein, similar to what happened with the novo proteome during memory formation [12]. The molecular identity of synaptic tag components and PRPs is still not well understood, although several candidates have been proposed [37]. This ‘synaptic-tagging and capture’ hypothesis establishes a framework to understand synaptic plasticity events happening during learning. However, whether synaptic plasticity and LTP are the mechanisms responsible for memory formation or just correlate with the process of learning is not yet elucidated, with recent results that either reinforced or weakened the SCT hypothesis [1]. Besides, memory-related epigenetics and transcription (that are cell-specific mechanisms) need to be included in an essentially synapse-specific scheme [1].

Given that memories can be stored for a long time (years in the case of mammals), the maintenance of reinforced synapses and modified cellular properties should be achieved by a specific cAMP response element-binding (CREB)-dependent transcriptional program [28]. In fact, it is well established that *CREB* and cAMP signaling are essential to form LTM in virtually all animal species [30]. Biological molecules (including proteins) are not immune to molecular turnover, with the exception of DNA. However, a prion-like self-replicative system could theoretically maintain LTM for a prolonged period of time without the need of transcription [44]. Such molecule might have been identified as orb2, an amyloid CPEB protein that linked memory formation and consolidation at least in the fruit fly [32] (see below for a discussion). The main argument favoring the role of transcription in memory is that protein translation is mandatory in order to create LTM [45, 54]. This is true for virtually any LTM formation, with the exception of a particular context-dependent LTM in *Drosophila* [60].

A way to keep these changes fixed is through epigenetics, which is defined as ‘the study of changes in gene function that are mitotically and/or meiotically heritable and that do not entail a change in DNA sequence.’ [56]. Francis Crick postulated that memory might be recorded as reversible modifications of DNA and proteins regulated by an enzymatic mechanism [10]. Modern epigenetics includes histone and non-coding DNA modifications, chromatin structure and non-coding RNAs [29]. In recent years, the continuous development of new sequencing techniques has provided increasingly detailed studies on epigenetic changes that could sustain memory formation, in addition to transcriptional responses [14]. Indeed, chromatin architecture and epigenetic signatures change dramatically after neuronal activation in at least two areas of the mouse brain such as the hippocampus and the cerebellar vermis [13, 58]. A comprehensive merged landscape of chromatin accessibility, epigenetic markers and transcriptional activity has been recently proposed [34]. Ultimately, such epigenetic modifications are aimed to maintain a specific transcriptional program(s) triggered after the experience that is required to create the cellular engram.

Our aim here is to summarize recent findings regarding the study of transcriptional response to memory formation in mouse to discuss the possible existence of a general transcriptional signature for memory, evaluating evidences and caveats.

## STEPS OF MEMORY FORMATION

What follows is a very brief and over-simplified view of the most accepted hypothesis regarding memory formation. The process of LTM comprises several loosely defined steps. In terms of transcription, experience (sensory + spatial + temporal information) triggers an immediate early response gene transcriptional wave (IEGs) that does not rely on new protein synthesis but depends on CREB activity (Fig. 1). It should be highlighted that the vast majority of CREB-regulated genes are not IEGs [24]. CREB plays several roles in many processes beyond memory (such as cell survival, proliferation, development, metabolism and neuronal plasticity) [4, 36].

These IEGs, in turn, activate a second surge of late-response genes (LRGs) [18]. Until recently, the consensus view was that there was a common set of IEGs that induces differential expression of LRGs, depending on the neuronal subtype [47]. Among them, *c-fos*, *arc, egr1* and *Npas4* are IEGs that are almost always highly expressed in at least part of experience-activated neurons (see [28] for a complete picture). However, single-cell transcriptomics experiments challenged this idea. Indeed, the current view is that distinct paradigms and cell types trigger diverse IEG responses, although the core is composed of classical bona-fide IEGs [23, 57]. How these IEGs can control the circuit reorganization is starting to be dilucidated. For instance, after spatial exploration, Scg2-mediated *fos* IEG activity triggers a bidirectional change in inhibitory circuits impinging on the hippocampal principal neurons [59].

In the following 24 hours after experience, consolidation takes place, where some neurons of the cellular ensemble reinforce their synaptic contacts [26]. This requires *de novo* protein synthesis and probably transcription (Fig. 1). The second presentation of the same stimulus that elicited memory formation causes the memory recall: only a subset of the cellular engram fires together to reproduce the sensorial experience, thus forming the memory engram. Besides, the stimulus induces the activation of other neurons, not previously included in the engram, which modify memory itself (Fig. 1). Memory storage can last for months or even years, indicating that a transcriptional and probably epigenetic program is set up to maintain the memory engram in time, in order to allow the fast and, to some extent, faithful retrieval. Some neurons of the cortical ensemble (located in the mammalian cerebral cortex) generated through a visual stimulus, maintain their activity up to 45 days, suggesting that they may constitute the substrate for a memory ensemble [38].

**Figure 2:**
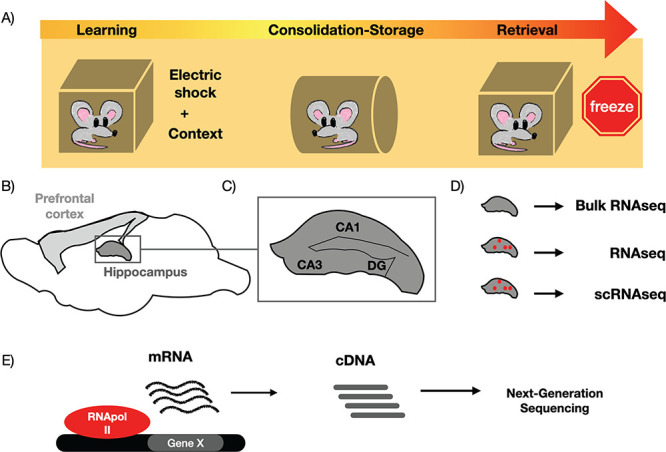
Description of experimental approach to study the transcriptional memory trace. (**A**) Fear conditioning implies an electric shock associated to a particular context. After consolidation, under the same context the mouse freezes. (**B**) The main structures where transcriptomics has been employed to address memory were the hippocampus and the prefrontal cortex. (**C**) CA1, CA3 and DG are regions of the hippocampus. (**D**) Bulk *RNAseq* does not distinguish at the cellular level. Marking either a specific population or activated neurons is able to reduce background noises. *scRNAseq* allows an accurate description of mRNA content for each cell. (**E**) *RNAseq* creates *cDNA* of thousands of *mRNAs* that are sequenced by next-generation techniques, even from one single cell.

Another intriguing question is the process known as systems consolidation [53]. As time goes by, the importance of engrams located in different structures for memory retrieval change and reorganization takes place. For instance, in mammals, the hippocampus, amygdala and other subcortical areas are essential for memory retrieval within the first week after the experience. After a month, these structures seem dispensable for memory storage, whereas the neocortex becomes critical to retrieve the experience (Figs 1 and 2). Intriguingly, 24 hours after a unique memory event, engrams in many different brain regions were identified, including several cortical structures [42]. Indeed, engram cells of the prefrontal cortex appear during learning, although they only become active during remote recall [31]. Whether the hippocampus is always required for memory retrieval or not is nevertheless under debate, although it is true that the hippocampal engram becomes silent two weeks after learning [31, 51]. How it is achieved at the molecular and transcriptional level is still largely enigmatic.

## HOW TO STUDY THE TRANSCRIPTIONAL MEMORY TRACE

The experimental design to study memory needs to consider the type of memory to be tackled i.e. the kind of learning paradigm to be used. The most employed memory test, by far, is fear conditioning (FC), a classical aversive associative assay (Fig. 2). The mouse associates a particular context (conditional stimulus—CS) to a mild electric shock (unconditional stimulus—US). After the consolidation period, a re-exposure to the same context causes a freeze fear response that can be measured. Practically all experiments concerning transcription and LTM in mammals (i.e. mouse) have been performed using this paradigm, which might render a biased view towards associative learning and negative experiences. We know that circuits, structures and engrams are different depending on the learning paradigm [53]. It is conceivable that memory-associated transcriptional changes may also be distinct. Currently, a high variety of learning and memory tests are available in mouse [15], and, together with new developed transcriptomic techniques, may give very insightful information regarding the molecular machinery involved and the existence (or not) of common features.

The second aspect to contemplate is the brain structure where we want to study these transcriptional changes, i.e. where the engram is expected to be allocated. In mouse, the vast majority of studies focused on the hippocampus, a structure that is necessary to form and store new associative memories (such as FC) (Fig. 2B). However, after a few weeks, it is dispensable and the cerebral prefrontal cortex becomes essential [31]. The hippocampus contains CA1, CA3 and dentate gyrus (DG) regions, but the experimental design does not allow us to distinguish between different regions.

A third feature is the type of RNA sequencing (RNAseq) employed to identify such transcriptional changes (Fig. 2). Classically, the transcriptional signature of tissues and structures was obtained by pooling all individual transcriptomic data together, without discriminating individual cell-type responses (i.e. bulk RNAseq). More recently, new Green Fluorescent Protein (GFP)-derived tools tools have allowed the discrimination among particular cell populations but, again, mixing up the mRNA of all neurons. Both techniques have been extensively employed to characterize the transcriptional correlates to LTM mainly in the hippocampus [43]. Currently, however, they have been progressively substituted by single-cell transcriptomic techniques such as scRNAseq (single-cell RNAseq) [7]. They take advantage of IEG-based transgenic constructs that permit to mark activated neurons after a particular experience, thus greatly reducing the noise due to the non-memory background activated cells [14].

The main technique used to identify the whole *mRNA* of a tissue or a single cell follows a simple scheme, basically identical to a classical qPCR experiment but without targeting a particular gene and for many mRNAs [49]. After extracting *mRNA*, a complementary DNA library is prepared by using a reverse transcriptase (Fig. 2). Single-cell transcriptomics approaches have evolved simultaneously to next-generation sequencing techniques, which allowed us to detect up to millions of mRNA-derived cDNA copies [49].

## UP-TO-DATE TRANSCRIPTIONAL STUDIES OF ACTIVATED (MEMORY?) NEURONS

Many studies used bulk *RNAseq* in whole tissue (or for a particular neuronal subpopulation) in order to identify the transcriptional response to a particular experience and memory formation and have been reviewed elsewhere ([6], [43]). Given the low number of neurons that are activated by a specific experience, resolution was far from ideal. The recent development of IEG-derived GFP sensors permitted the labeling of activated engram neurons solely, which, combined with scRNAseq, allowed to increase greatly the resolution towards the cellular engram that include the memory engram. But, how to distinguish ‘memory’ from ‘experience’ cells, given that both are linked to the intrinsic transient nature of IEG expression? [23]. One possible strategy is to employ two different IEG markers, either with different decay timing, or using a genetically traceable marker [8, 25, 35]. In the latter case, a *fos*-dependent TRAP transgenic line marks permanently the neuronal activity in the experience engram (plus classical IEG labelling such *arc* or *fos* antibodies) or exclusively in the reactivated memory engram [35]. In this way, retrieval and storage can be studied independently. Here, we focus on recent studies that have tried to describe the neuronal transcriptional response to memory formation at a single-cell resolution.

Novelty exploration paradigm takes advantage of the exploratory behavior of mice when immersed in a cage with new cues. This assay was used to determine the specific early transcriptional signature in the hippocampus after the experience and the response when confronted with the same cage [25]. Interestingly, most transcriptional experience-induced changes occurred in DG cells. There was some overlapping in the response between different neuronal populations, but data pointed towards a cell-type specific transcriptional response. Besides, due to a differential decay of Fos and Arc proteins, the authors could identify the transcriptional response of DG neurons both, before and after reactivation. In an early phase, this response contained genes that modified the epigenetic and transcriptional state. In contrast, some LRGs were related to neurite outgrowth and secreted extracellular matrix proteins. The analysis also showed a transcriptional signature that identifies, unequivocally, DG neurons that are going to be reactivated by the re-exposure to the environment. The interpretation of results emanated from this paradigm is not obvious. The animal compared the new environment with one already known in order to find differences using several brain regions, a process known as memory recognition [52]. So, the hippocampal reactivated network might reflect a novelty-coding circuit, the habituation process or even semantic-memory formation, among other possibilities [11, 27, 52]. The authors suggested that the transcriptional signature might be related with selecting the components of the stimulus-activated engram that were involved in memory encoding or retrieval [25].

In another study, 24 hours after applying FC, the DG engram neurons were studied using targeted recombination in active populations (fos-TRAP) [39]. The vast majority of differentially expressed genes had not been identified in previous studies, with enrichment in ‘receptor binding’ and ‘ion channel activity’ genes. Interestingly, almost half of up-regulated genes were under CREB regulation and in fact, consolidation required a CREB-dependent transcriptional response. Again, a transcriptional signature was extracted that was unique to these types of engram cells.

A similar experimental approach was employed more recently, but considering two additional features: memory-associated chromatin changes and the recall response [35]. The main finding of this paper is that memory encoding caused an increase in chromatin accessibility that was not reflected in transcription, whereas consolidation phase was characterized by changes of promotor–enhancer interactions. Focusing on transcription, there was a stable cluster of genes related to cytoskeleton and axon remodeling that was up-regulated following experience and maintained during all memory stages. Regarding memory recall, a reactivation gene cluster (2 hours after the re-exposure) was mostly related to RNA transport and translation. They found weak overlapping with previous data. Intriguingly, recent advances in proteomics allowed us to identify about 150 proteins with altered synthesis during spatial LTM formation, with functions related to mRNA splicing, vesicle-mediated transport and Rho GTPase effectors, among others [12]. Unfortunately, the technique did not allow us to distinguish between different memory phases, and also the memory paradigm employed was different from FC [12].

In the last study, the prefrontal cortex cells (by means of an improved Cre-dependent *fos*-TRAP2 activated during retrieval) were interrogated 10 days after re-exposure and 25 days after learning, thus looking for memory storage [8]. Three interesting ideas emerged from that study. First, there were long-term associated changes in glial cells, especially related to metabolism (astrocytes) and cytoskeleton/immunity (microglia), something that suggested their potential role in memory storage. Second, there was an enrichment of vesicle exocytosis and transport-related genes. Lastly, no significant overlapping in terms of genes appeared with previous results, maybe indicating that remote memory might be regulated by temporally unique transcriptional programmes.

## DRAWBACKS AND LIMITATIONS

Given the complexity of the process, the transcriptional study of LTM formation and retrieval shows important limitations. In mammals, one important drawback is the identification of the cell population of the engram. The strategy is to mark activated cells by immediate early response genes such as *fos, arc* or a combination of both. Therefore, there would be a possible misrepresentation (or bias) of the cell number and type: it has been described that at least two different neuronal ensembles with specific functions may co-exist within the same memory engram, using two different IEGs such as *fos* and *npas4* simultaneously [50]. Furthermore, the early transcriptional response with similar labeling methods, but under different IEG control (*fos* vs *arc*), shows little overlapping, thus indicating again that some experience engram cells are missing [8, 25, 35].

The bioinformatic analysis is also an important point to consider. Positive and negative controls must be carefully chosen to minimize the noise caused by other inputs that can dilute the real response [49]. Before and after training (and in later stages as well), temporal transcriptional changes of the same neurons are monitored. As a consequence, sequencing only thousands of mRNAs may not uncover underrepresented mRNAs, thus demanding deeper sequencing. Furthermore, there are no clear clustering and differential expression guidelines given that transcriptional differences appear between very similar neurons. The biological importance of particular genes (or gene classes) is not contemplated in the algorithms. Novel pipeline scripts and more efficient tools are needed urgently.

The spatial aspect of memory is another pitfall of these approaches. Most studies focus in one particular structure such as the hippocampus or the prefrontal cortex. The current view is built on the idea that a circuitry comprising different areas of the brain is necessary for memory formation, consolidation and retrieval, which was not assessed simultaneously in published transcriptional studies [9, 42]. Also, different neuronal subtypes participate in a single memory event, thus adding complexity. Single-cell spatial transcriptomics is trying to solve this issue, although that technology requires still further development [2].

But perhaps the most important caveat is the temporal aspect of the problem. The single-cell transcriptomic technology takes a very detailed picture of the transcriptional landscape of several neurons, but at a particular time point. Memory consolidation, storage and systems consolidation are, likely, gradual processes that require hours, days or even weeks. If it is progressive, we need to define stages that imply to follow the temporal development of transcriptional changes for several genes. In fact, the phases in which we divide the process of memory formation are clearly arbitrary. For instance, is it possible to identify the particular moment in which a memory is fully consolidated or stored? The key point may rely in the intrinsic dynamic nature of memory and its engrams. In order to understand the different stages of memory, a transcriptional movie will render invaluable information. Obviously, such approach is currently beyond the possibilities of the available technology.

## DOES A TRANSCRIPTIONAL TRACE OF MEMORY EXIST?

We are barely starting to identify the transcriptional repertoire that memory formation elicits thanks to the new developed NGS and bioinformatics techniques. We are still far from understanding the organizing principles due to the high complexity and dynamic nature of the process. Maybe, due to such complexity, current data support the view that the transcriptional response seems to be specific for each particular neuronal and/or memory type. However, a general, conserved transcriptional signature that identifies unequivocally neurons that participate in LTM formation may exist. Actually, this is the case for CREB/cAMP signaling that play a conserved role in memory formation across the animal kingdom, although it has other functions [36]. But how to identify such transcriptional memory trace?

An important aspect to discuss first is what stage of memory formation is more susceptible of having a shared memory mark. Consolidation seems to be out of the question, given the uncertainty of process duration and molecular mechanisms involved. It is not clear when it starts and ends, and which would be the most adequate time point to study. On the other hand, learning, retrieval and storage remain as more appropriate options.

The learning process is a clear candidate, given the importance of CREB phosphorylation for encoding LTM [28]. Indeed, current view supports the idea that different experience-activated neuronal populations might share a small core of IEGs, despite most IEGs are cell-type specific [23, 57]. Still, how many of these common IEGs co-express simultaneously in the same activated neuron or if the complete engram can be uncovered by a specific transcriptional fingerprint is still not known [8, 25, 35, 50]. An alternative possibility comes from the fact that neuronal activity related to memory formation is accompanied by a phenomenon of neuronal plasticity, such as synapsis reinforcement (i.e. synaptic plasticity), modifications in neuronal excitability or even re-wiring [16, 40]. All these processes would require a transcriptional programme mainly regulated by IEGs. In this way, the transcriptional signature that identifies memory formation might be characteristic for each neuronal subtype and focused on IEG-dependent neuronal plasticity genes, together with a core of common IEGs. The complexity of the gene expression pattern might be alleviated by using exclusively up-regulated genes related to changes in synaptic plasticity (that might include PRPs). However, to distinguish between neuronal and memory-only engram cells is still challenging and it might not be feasible using uniquely IEGs or synaptic plasticity-related genes. Also, the IEG response might be specific of the type of memory generated in addition to the neuronal sub-type, which would imply an additional level of complexity.

During retrieval, engram neurons specifically up-regulate a specific set of genes 2 hours after re-exposure [35]. These genes are apparently unspecific, so the question is whether or not some of them are also present during retrieval in other types of learning, brain structures or even organisms. The nature of such genes is mainly related to RNA transport/binding and protein translational mechanisms [35]. Intriguingly, these results support the hypothesis of prion-like proteins, based mainly on *Drosophila* results: it has been postulated that some RNA-binding proteins (CBEP/orb2) with a functional prion-like state might be a molecular substrate of memory [21, 46]. However, *orb2* is not required in all *Drosophila* learning paradigms [17], suggesting either different mechanisms for distinct types of memory or that other RNA-binding proteins still remain to be described.

Storage is the other plausible stage to look into. Two clear advantages are the stability in time, suggesting a very stable molecular transcriptional machinery, and a moderately good correlation between epigenetic markers and active transcriptional sites both in *Drosophila* and mouse [19, 22]. Classically, an important problem in epigenetic studies is that transcriptions do not usually match with active chromatin and vice versa when studied simultaneously [33]. However, this was probably because, as a first step, epigenetic marks are set and a few hours/days later transcription changes reveal according to the chromatin state previously established [34]. Unfortunately, scRNAseq studies from LTM storage structures (like the prefrontal cortex) came only from FC, showing an enrichment of genes related to vesicle-mediated transport, exocytosis and neurotransmitter secretion [8]. Again, the general character of such molecules is a strong and a weak point, simultaneously. A possible mechanism to maintain memory with this transcriptional repertoire might be linked with synaptic plasticity and LTP. However, the cortex is important for memory storage after systems consolidation. Thus, one may wonder if some of these genetic players also appear in other memory-related structures such as the hippocampus or the cortex employing a different learning paradigm.

In summary, recent technical advances in molecular tools, transcriptomics and bioinformatics allowed us to take a first glimpse to the transcriptional response in engram cells during memory formation, retrieval and storage. Huge efforts have been done despite limitations of current techniques. Altogether, data suggest that most of transcriptional changes are cell- and memory type-specific, although we propose some hints to find the still elusive transcriptional trace of memory. Comparing data from different types of memories and animal models might be a sensible approach in order to find a common pattern.

## SUPPLEMENTARY MATERIAL

Supplementary material are available at *Oxford Open Neuroscience* online.

## FUNDING

This work was supported by the Spanish Research Agency (MICINN) under the grant PGC2018-094630-B-100 to FAM,cofinanced by the European Regional Development Fund (ERDF). F.A.M. is a recipient of a RyC-2014-14961 contract. B.G.-M. is a recipient of a predoctoral fellowship, grant number SFPI/2020/00878 (UAM). C.G.B. is a recipient of a FPU predoctoral fellowship, grant number FPU19/04449 (MEFP). S.P.-F. is a recipient of a JAE intro fellowship, grant number JAEINT_21_02520 (CSIC).

## AUTHORS’ CONTRIBUTIONS

B.G.-M., C.G.B., S.P.F., E.T. and F.A.M. are responsible for writing the original draft. F.A.M. is responsible for the conceptualization, funding acquisition and project administration. All authors contributed to the article and approved the submitted version.

## DATA AVAILABILITY STATEMENT

No data included in this manuscript.

## ETHICAL STATEMENT

No ethical requirements for this manuscript.

## CONFLICT OF INTEREST STATEMENT

The authors declare that they have no conflict of interest.

## Supplementary Material

suppl_data_kvac008
