# Supplementary material for: The elusive transcriptional memory trace
Source: Oxf Open Neurosci. 2022 Jun 16;1:kvac008. doi: 10.1093/oons/kvac008 (PMC10913820; doi:10.1093/oons/kvac008)
Supplement: suppl_data_kvac008 [file suppl_data_kvac008.zip › kvac008_Reviews_for_transparent_peer_review kvac008.docx]

Reviews: The elusive transcriptional memory trace

Original manuscript:

Reviewer 1 - The authors attempt to summarize the literature and concepts regarding the relationship between gene transcription and the possible formation of memory engrams. Indeed the topic is rather difficult to address due to the multitude of memory types and experimental models utilized to study memory formation. In addition, the terms regarding memory formation need to be clearly defined because of the different aspects of physiology and psychology. In this respect, the review would benefit to discuss whether IEGs are suitable tools in the search of memory traces and whether there is a common transcriptional pattern in some aspects of memory formation. Therefore the authors could consider focusing on specific memory models and highlight the current knowledge regarding transcriptional regulation in response to learning in such models. At the moment the review reads like a collection of ideas without clear details, which makes it difficult to get a take-home message.

Figures.

Is IEG expression indeed CREB mediates? Most CREB regulated genes are not IEGs. Since CREB phosphorylation is a hallmark for long-term memory, the authors might discuss if IEGs are good markers for memory formation. Many genes that are required to “reconstruct” synapses, neuronal excitability, or even “rewiring” are regulated by a multitude of transcription factors, making it possible to have a “used-dependent” expression pattern. Since literature references are missing, it is difficult to distinguish between experimentally proven statements and ideas. At least from one of Tonegawa’s papers, the conclusion was that the prefrontal cortex and hippocampus take part in learning and consolidation at the same time, but that the impact of the hippocampus for memory recognition is fading with time.

The novelty experiments are based on the differentiation of the environment with previously stored information (memory). Thus, the sensation of an environment activates neuronal ensembles that need to be discriminated as never before experienced or well known – a process that requires specific brain regions. In this respect, the literature also differentiates between recognition memory and recall – the memory recall retrieves information that might be linked to different aspects of behavioral performance.

The authors need to check carefully the terms used to describe different aspects of memory and to focus on a small set of experimental models.

Reviewer 2 - This review mainly focused on data from Drosophila works. The role of transcription and translation is well established for LTM persistence. The review will largely benefit if the authors can expand description more from vertebrate’s neuronal system, specifically including protein synthesis data for associative plasticity/memory/synaptic tagging and capture etc.

Specific points:

Abstract: The sequential order should be: Encoding or learning, consolidation, Storage and recall/re-activation.

Figure1. Legend (line 391-92). : “After learning, neuronal activity ceases”. This is not true. According to the Hebbian associativity, there is always reverberating changes occurring in the activated neural networks.

Revised version

Reviewer 1 – ACCEPT

Reviewer 2 - The review draws a comprehensive picture of the transcriptional regulation in engram neurons. The authors focus on a few explorative memory models commonly applied in mice and indicate the relationship with mechanisms of protein turnover regulation in activity-dependent synaptic plasticity. In addition, the authors review current technologies that target the transcriptome in engram neurons. The review helps to recognize conceptual problems in memory research and introduces a basic model of memory-related transcriptional regulation.
